# VP-SFDA: Visual Prompt Source-Free Domain Adaptation for Cross-Modal Medical Image

**DOI:** 10.34133/hds.0143

**Published:** 2025-01-07

**Authors:** Yixin Chen, Yan Wang, Zhaoheng Xie

**Affiliations:** ^1^ Institute of Medical Technology and National Biomedical Imaging Center, Peking University, Beijing 100191, China.; ^2^ School of Instrumentation and Optoelectronic Engineering, and State Key Laboratory of Software Development Environment, Beihang University, Beijing 100191, China.

## Abstract

**Background:** Source-free unsupervised domain adaptation (SFUDA) methods aim to address the challenge of domain shift while preserving data privacy. Existing SFUDA approaches construct reliable and confident pseudo-labels for target-domain data through denoising methods, thereby guiding the training of the target-domain model. The effectiveness of denoising approaches is influenced by the degree of domain gap between the source and target domains. A marked shift can cause the pseudo-labels to be unreliable, even after applying denoising. **Methods:** We propose a novel 2-stage framework for SFUDA called visual prompt source-free domain adaptation (VP-SFDA). We propose input-specific visual prompt in the first stage, prompting process, which bridges the target-domain data to source-domain distribution. Our method utilizes visual prompts and batch normalization constraint to enable the alignment model to learn domain-specific knowledge and align the target-domain data with the source-domain contribution. The second stage is the adaptation process, which aims at optimizing the segmentation model from the source domain to the target domain. This is accomplished through the denoising techniques, ultimately enhancing the performance. **Results:** Our study presents a comparative analysis of several SFUDA techniques in the VP-SFDA framework across 4 tasks: abdominal magnetic resonance imaging (MRI) to computed tomography (CT), abdominal CT to MRI, cardiac MRI to CT, and cardiac CT to MRI. Notably, in the abdominal MRI to CT adaptation task, the VP-OS method achieved a remarkable improvement, increasing the average DICE score from 0.658 to 0.773 (*P* < 0.01) and reducing the average surface distance (ASD) from 3.489 to 2.961 (*P* < 0.01). Similarly, the VP-LD and VP-DPL methods also showed significant improvements over their base algorithms in both abdominal and cardiac MRI to CT tasks. **Conclusions:** This paper proposes VP-SFDA, a novel 2-stage framework for SFUDA in medical imaging, which achieves superior performance through input-specific visual prompts and batch normalization constraint for domain adaptation, coupled with denoising methods for enhanced results. Comparative experiments on 4 medical SFUDA tasks demonstrate that VO-SFDA surpasses existing methods, with ablation studies confirming the benefits of domain-specific patterns.

## Introduction

Deep convolutional neural networks (DCNNs) have demonstrated remarkable success in various computer vision tasks, particularly in medical image analysis [1–6]. However, real-world medical applications present marked challenges due to the variability in data sources, such as differences in imaging modalities or variations across hospitals. Manually annotating data to account for these variations is both time-consuming and cost-prohibitive. To address these challenges, source-free unsupervised domain adaptation (SFUDA) offers a promising solution, by enabling models to adapt to new domains without requiring access to source data. This approach offers several critical benefits: It reduces annotation costs through cross-domain knowledge transfer, enhances privacy by eliminating the need to share source data, and maintains model performance across diverse clinical environments.

For instance, SFUDA allows models trained on computed tomography (CT) scans at one hospital to adapt to magnetic resonance imaging (MRI) scans at another without accessing the original CT data, safeguarding patient confidentiality. This is particularly valuable in resource-limited regions, where models can adapt to varying devices and patient demographics, improving diagnostic accuracy despite limited standardized equipment and datasets. Additionally, SFUDA facilitates seamless model adaptation across populations in international collaborations, where data privacy regulations restrict patient data sharing. With the rise of mobile medical devices generating diverse data, SFUDA ensures consistent model performance across different clinical environments.

Most current research in domain adaptation has focused primarily on addressing the technical challenges of adaptation, often overlooking the equally critical aspect of data privacy [7–13]. Unlike traditional unsupervised domain adaptation methods, which require simultaneous access to both source and target data, the method explored in this study adheres to the SFUDA paradigm, offering a more secure and efficient pathway to achieving domain adaptation in real-world clinical scenarios.

**Fig. 1.**
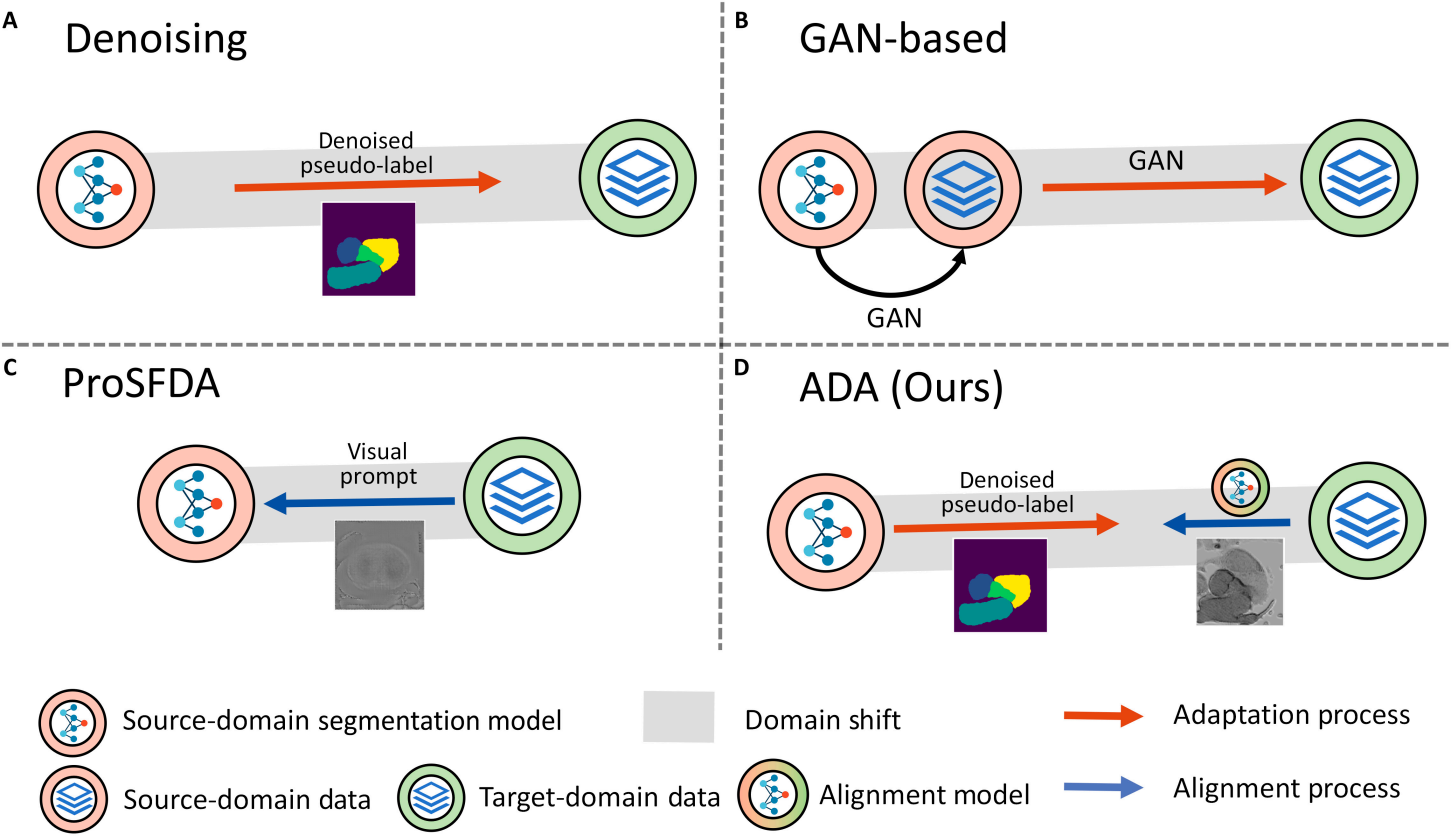
Different solutions for SFUDA tasks. (A), (B), and (D) focus on the tasks with different modalities (large domain shift). GAN-based methods are used in natural image scenario. The approach in (C) handles the task with different medical centers (small domain shift).

Figure 1 shows a variety of solutions for SFUDA. In Fig. 1A, we depict an approach that utilizes denoising of pseudo-labels for enhancing the adaptability of models trained on a source domain to perform efficiently on a target domain. Given the inherently noisy nature of pseudo-labels generated for the target domain by models trained on the source domain, it is crucial that the noise in pseudo-labels can be mitigated to produce more reliable labels. However, the extent of the domain gap presents a important challenge. A larger gap results in noisier pseudo-labels, undermining the efficacy of the adaptation process. Figure 1B illustrates a method that employs generative adversarial network (GAN) to restore inaccessible source-domain data, followed by the application of GANs again to facilitate domain adaptation. While this method has shown some effectiveness in the context of natural images, it is not without its challenges. The integration of GANs introduces an element of instability in the training process. This instability becomes particularly pronounced in medical imaging tasks, where the availability of data is often limited. Consequently, the performance of this method can be compromised in these scenarios, undermining its reliability and efficacy for domain adaptation in medical imaging. Figure 1C represents another strategy that employs input-agnostic visual prompts for aligning domains. Our proposed method incorporates “prompting” and “denoising” phases, as shown in Fig. 1D. We employ visual prompts to bridge the domain disparity, yielding substantially enhanced adaptation outcomes.

### Visual prompting

Prompting fine-tunes pretrained language models for downstream tasks by adding language instructions to input text, enhancing task understanding without altering model parameters [14,15]. This approach extends to computer vision, where vision-language models align images and text in a shared embedding space or use prompts in vision tasks without text. Vision-language models, initially explored nearly a decade ago [16–18], have advanced recently with multi-modal models [19–23], such as CLIP [23] and ALIGN [24], which leverage contrastive learning for text-image alignment. However, these models require extensive datasets—CLIP with 400 million pairs and ALIGN with 1.8 billion pairs—making them less feasible for medical imaging due to limited large-scale, high-quality text-image pairs. To address this, we introduce the concept of visual prompting into medical imaging. Visual prompting alters the behavior of pretrained models without changing their parameters [25,26], designing specific prompts for different tasks. It has been demonstrated that visual prompting outperforms text prompting in image classification and is robust to distribution shifts [25]. Recent work, such as FVP [27], explores using input-agnostic visual prompts in Fourier space to tackle SFUDA tasks.

### Batch normalization

DeepInversion [28] is a very enlightening research. They aim to restore the training data from a pretrained model. The mean and variance stored in the batch normalization layers of the pretrained model are the source-domain-specific statistics. Batch normalization (BN) adjusts the mean and variance of feature maps to account for statistical variations between different datasets [29]. Furthermore, Santurkar et al. [30] provide insights into how BN layers adapt to different data distributions during training, thereby inherently characterizing the dataset’s features. Therefore, the means and variances of BN layers could represent characteristics of the source-domain data. By aligning these statistical properties with those of the target domain, our method effectively mitigates domain discrepancies, thereby enhancing model generalization across different datasets.

We introduced visual prompt source-free domain adaptation (VP-SFDA), which embodies 2 stages. In the first stage, we merge batch normalization constraint (BNC) with visual prompts, enhancing stability during the domain adaptation of images. This approach specifically targets image-wise adaptation by reducing domain shift. The second stage focuses on model-wise adaptation, where denoising of pseudo-labels enhances the source model’s generalization capabilities. By sequentially addressing both image-wise and model-wise adaptations, our 2-stage SFUDA framework offers a more comprehensive solution compared to traditional single-stage adaptation methods. Additionally, it is essential to note that previous methods referenced as [25,27,31] adopt input-agnostic visual prompts. This term signifies a uniform application of a single prompt pattern across the entire dataset, treating all inputs identically without considering the specific characteristics or variations among individual data samples. This “one-size-fits-all” approach, while effective in some contexts, can be limited in its adaptability and performance during the domain adaptation process. In contrast, input-specific visual prompts are tailored to each distinct input. Each data sample, with its unique features and characteristics, is addressed individually, ensuring a more nuanced and adaptive approach.

## Methods

We propose a 2-stage SFUDA framework, VP-SFDA. The first stage (prompting) shortens the domain shift to improve the quality of pseudo-label. We design an alignment model (AM) to generate prompts for target-domain images. A well-trained AM creates the input-specific prompt. The performance of the target-domain data degrades on the source model, while merging the target-domain data and prompts can effectively avoid this dilemma. The pseudo-label of the prompted target-domain data contains less noise. In the second stage (adaptation), we could utilize the pseudo-label and denoising methods to update the source model.

Our 2-stage framework leveraging denoised pseudo-labels offers distinct advantages over methods relying solely on visual prompts. First, our approach inherently includes the visual prompting process; hence, even in scenarios where denoised pseudo-labels might be less effective, our model is capable of achieving at least equivalent performance to methods that utilize only visual prompts. Second, methods like ProSFDA [31] that do not modify the source model inherently suffer from poor robustness. Such model only trained on source-domain data, making them susceptible to performance degradation when facing input data with variations. In contrast, our method not only utilizes visual prompts to bridge domain discrepancies but also enhances the source model’s robustness through the fine-tuning process with denoised pseudo-labels. This fine-tuning involves data from both the source and target domains, thereby enriching the training dataset and subsequently enhancing the model’s robustness.

### SFUDA settings

In the SFUDA setting, we are given a segmentation model, denoted as $f^{s}:X^{s}\to Y^{s}$, which is trained on a source domain $D^{s}=\left(X^{s},Y^{s}\right)$. Concurrently, we have an unlabeled dataset from the target domain, represented as $D^{t}=\left(X^{t}\right)$. The well-trained source model, $f^{s}$, is capable of rendering accurate inferences on the data $X^{s}$ from the source domain. The core objective of the SFUDA task is to derive the target model $f^{t}$ in scenarios where the source domain $D^{s}$ is inaccessible. This adapted target model $f^{t}$ should ideally make effective inferences on the target-domain data $X^{t}$.

If source model $f^{s}$ is directly applied to the target-domain data $D^{t}$, there is huge performance degradation due to the domain gap. The goal of SFUDA is to avoid this performance degradation by only using the unlabeled target-domain data $D^{t}$ and the pretrained source model $f^{s}$. The setting of SFUDA can be applied to different specific tasks, such as classification and segmentation. Compared with the image-wise classification task, the segmentation task can be considered a more difficult pixel-wise classification task. In this paper, we focus on medical image segmentation, which is a multi-label segmentation problem with $x_{i}\in {\mathbb{R}}^{H\times W\times 1}$ and $y_{i}\in {\left\{0,1\right\}}^{H\times W\times C}$ (*C* denotes the class number), where $x_{i}\in X$ and $y_{i}\in Y$.

According to the setting of UDA researches [7] and SFUDA researches [32–34], the choice of source model is convolution network, which usually adopts DeepLabv3-ResNet50 [1]. Model $f^{s}$ could be optimized by minimizing the cross entropy, which is defined as $L_{\mathrm{CE}}$:

$$f^{s*}=\underset{f^{s}}{\text{argmin}}E_{x^{s}∼X^{s}}\left[L_{\mathrm{CE}}\left(f^{s}\left(x^{s}\right),\;y^{s}\right)\right],$$ (1)

where ${}^{*}$ represents the well-trained model.

### Input-specific pattern

In this section, we elucidate the importance of the input-specific pattern by tracing the motivation and methodological characteristics of 2 visual prompt patterns. The inception of visual prompts is traced back to Visual Prompt Tuning (VPT) [26], wherein they are portrayed as an efficacious methodology for fine-tuning large-scale transformer models in visual tasks. Further substantiation is found in [25], where the capability of visual prompts in transposing large-scale pretrained models to downstream CV tasks is empirically affirmed. These seminal works collectively underscore the instrumental role of visual prompts in augmenting the efficacy of large-scale pretrained models.

A notable implementation of visual prompts in SFUDA tasks is encapsulated in ProSFDA [31]. Its prowess is exemplified in the multi-domain joint optic cup and optic disc (OC/OD) segmentation task, while our analysis across multi-modal SFUDA tasks unveiled some limitations using ProSFDA framework. The constraints are attributed to the employment of input-agnostic patterns. We believe that input-agnostic pattern, albeit proficient in facilitating model transition to downstream tasks [25,26], are found wanting in multi-domain adaptability. Input-agnostic visual prompt has only been validated for effectiveness in 2-dimensional segmentation tasks of the OC/OD [31]. However, the adaptation challenges are greater in 3-dimensional segmentation tasks involving CT and MR data under unsupervised domain adaptation (UDA) settings. In this more complex scenarios, input-agnostic approach was ineffective. Conversely, input-specific visual prompt demonstrates enhanced adaptability.

Furthermore, input-agnostic visual prompts treat all inputs the same. They apply a uniform pattern or style to all incoming data. It might not capture the nuanced differences between individual data samples. As it is a “one-size-fits-all” method, it may not be optimized for specific characteristics of individual data samples. This can limit the adaptability and performance of the domain adaptation process.

In light of these observations, our manuscript introduces the concept of input-specific visual prompts, meticulously crafted to address the inherent limitations associated with the input-agnostic counterparts. These prompts are tailored to each specific input, and every CT image would have a unique visual prompt. Different data samples, especially in medical imaging, can exhibit a wide range of features, anomalies, and characteristics. This approach can cater to the individual characteristics and features of each data sample. Having input-specific prompts ensures that each data sample’s unique traits are considered, leading to a more nuanced and accurate adaptation.

### Alignment

#### Loss function

**Fig. 2.**
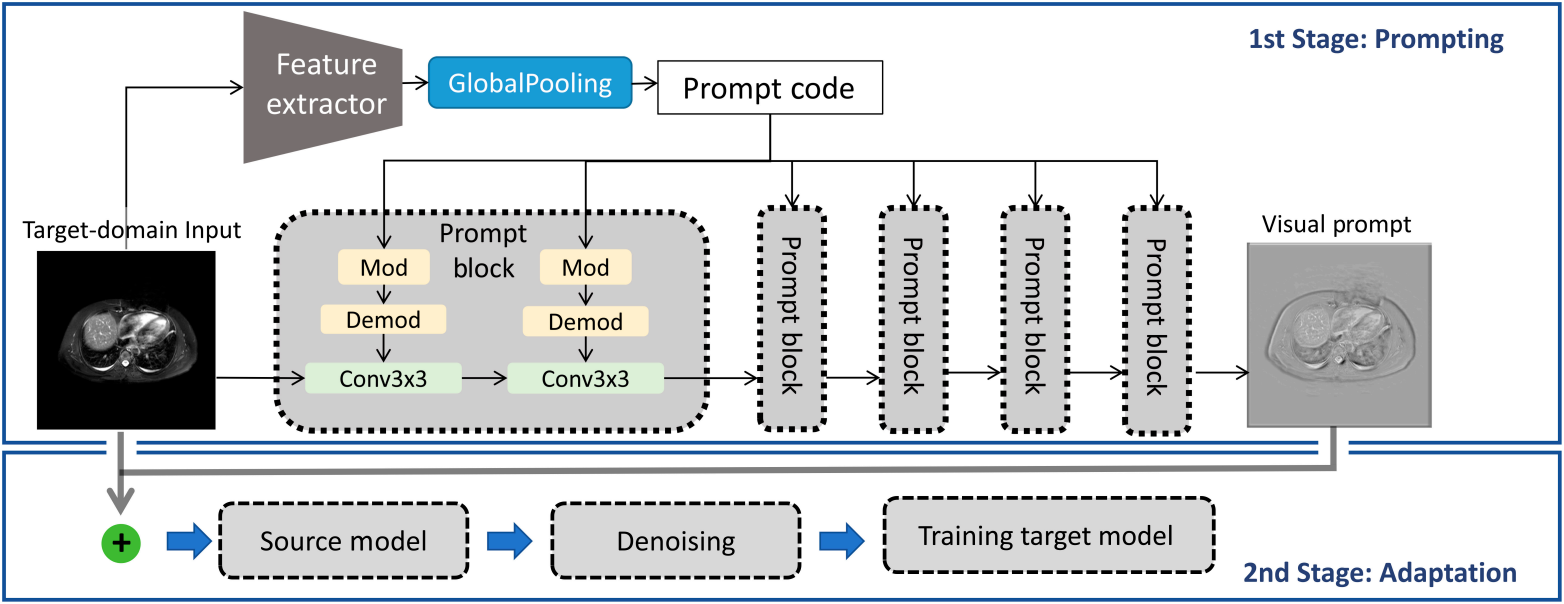
Structure diagram of VP-SFDA. The AM is a double-branch structure based on convolution in the prompting stage. The first branch extracts feature from images to obtain prompt code, and the second branch creates the input-specific visual prompt level by level under the guidance of prompt code. In the second stage, we could obtain pseudo-labels with less noise from various denoising methods.

We create an AM to generate prompts for the target-domain images. As shown in Fig. 2, AM can generate the corresponding visual prompts for the target-domain data so that the fused images of the target domain and the prompts can produce more accurate prediction results on the source model. In batch normalization, the mean ($\bar{\mu }$) and variance ($\bar{\sigma }$) are essential metrics representing the characteristics of the source-domain data. They are computed as running statistics across batches, capturing the dataset’s underlying distribution. The running mean and running variance not only are parameters but also serve as a compact representation of the source dataset’s characteristics. The parameters of AM are optimized by making the mean and variance of the feature maps of the target-domain image consistent with those stored in the batch normalization layer in the source model, which can be defined as:

$$\begin{array}{cc} L_{\mathrm{BNC}}= & E_{x^{t}∼X^{t}}\sum_{l}\left[{\left\|\mu_{l}\left(x^{t}+\mathrm{AM}\left(x^{t}\right)\right)-{\bar{\mu }}_{l}\right\|}_{2}+\right.\\ & \left.{\left\|\sigma_{l}\left(x^{t}+\mathrm{AM}\left(x^{t}\right)\right)-{\bar{\sigma }}_{l}\right\|}_{2}\right] \end{array}$$ (2)

where $x^{t}$ is the sample that obeys the target-domain distribution and $\mathrm{AM}\left(x^{t}\right)$ is the alignment prompt. ${\bar{\mu }}_{l}$ and ${\bar{\sigma }}_{l}$ are the source-domain mean and variance stored in the *l*th layer of the source model. $\mu_{l}\left(x^{t}+\mathrm{AM}\left(x^{t}\right)\right)$ and $\sigma_{l}\left(x^{t}+\mathrm{AM}\left(x^{t}\right)\right)$ are the batch-wise mean and variance of feature maps at the -lth layer.

**Fig. 3.**
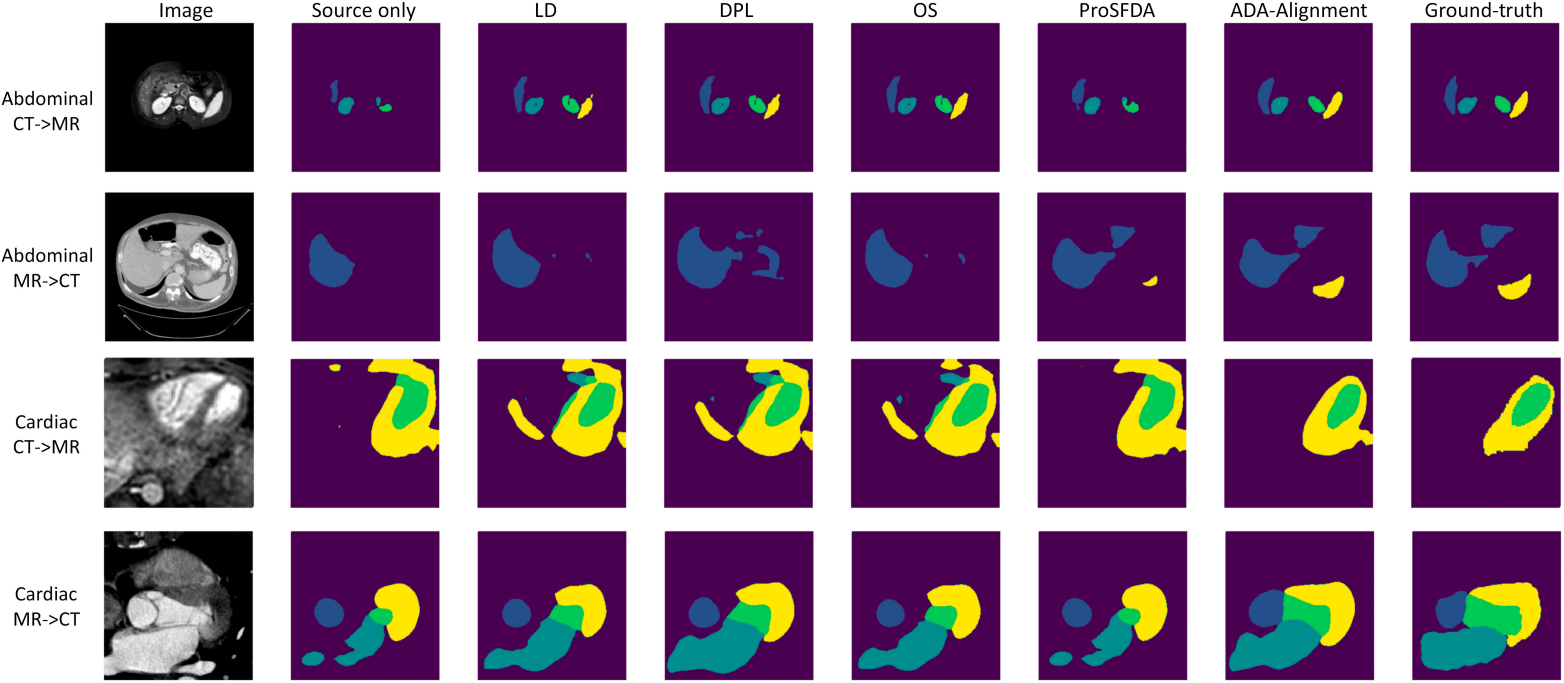
Visualization of prediction results. The first 2 rows are the segmentation results of the abdominal images. The third and fourth rows are 2 examples of cardiac images.

In Eq. 2, AM denotes the AM tasked with generating the input-specific prompt. Given that the parameters of our BN layers encapsulate the source domain’s traits, the mean and variance of the feature maps computed for the target-domain data, $x^{t}$, within the BN layers of the source model are anticipated to deviate. This discrepancy can be construed as a tangible manifestation of domain shift.

The reason of the input-specific visual prompt is to harmonize this disparity. It ensures that the prompted image, expressed as $x^{t}+\mathrm{AM}\left(x^{t}\right)$, yields mean and variance in the BN layers congruent with those of the source model. In essence, the visual prompt aligns the target-domain data with the source domain, mitigating the effects of domain shift.

To sum up, the intrinsic relationship between visual prompts and batch normalization lies in their concerted effort to bridge the gap instigated by domain disparities. Visual prompts facilitate the adaptation of the target-domain data, ensuring compatibility and coherence with the source domain’s BN layers’ parameters. Consequently, this synergy fosters enhanced performance and robustness in scenarios of domain adaptation.

#### AM design

Figure 2 shows the structural details of the AM. Since our prompts are input-specific, we need a model to generate the corresponding prompt for each image. The AM is a double branch convolutional neural network. As shown in Fig. 2, the first branch is to extract feature maps from images and obtain potential prompt code through the global pooling layer. The second branch is to convert the original image into the corresponding prompt step by step under the guidance of prompt code.

The AM has 5 prompt blocks, and each contains 2 convolution layers. Each layer consists of 3 × 3 convolutional kernels. The weights of the convolutional layers are initialized using the Kaiming He normal distribution, and the biases are initialized to zero. Each convolutional layer is followed by a leaky ReLU activation layer. The AM does not contain upsample or downsample layers. The general convolution can be defined as:

$$m^{l+1}=\text{conv}\left(m^{l}|\theta_{\mathrm{ijk}}^{l}\right),$$ (3)

where $m^{l}$ is the feature map at the lth layer. The feature map is the input image, when l=0. When l=10, the last layer feature map is the visual prompt $\mathrm{AM}\left(x^{t}\right)$. $\theta^{l}$ is the weights of the lth convolutional layer. i,j,andk are the channel numbers of $m^{l}$, channel numbers of $m^{l+1}$, and spatial footprint of the convolution kernel, respectively. Prompt code is defined as p that has a dimension of 1,024. Through a linear mapping, this prompt code is then linked to the space with i dimensions, denoted as $s_{i}$. Therefore, the combination of modulation and convolution can be:

$$m^{l+1}=\text{conv}\left(m^{l}|\theta_{\mathrm{ijk}}^{l}+s_{i}^{l}\right).$$ (4)

Modulation refers to the process of modifying the intermediate feature maps. However, removing the effect of $s_{i}$ from statistics of $m^{l+1}$ has proved to help generate high-quality images [35]. We assume the input activations are independent and identically distributed (i.i.d) random variables with unit standard deviation. We could use Eq. 5 to restore the output $m^{l+1}$ back to unit standard deviation.

$$m^{l+1}=\text{conv}\left(m^{l}|\frac{{\hat{\theta }}_{\mathrm{ijk}}^{l}}{\sqrt{\sum_{i,k}{\left({\hat{\theta }}_{\mathrm{ijk}}^{l}\right)}^{2}+\epsilon }}\right).$$ (5)

where ${\hat{\theta }}_{\mathrm{ijk}}^{l}$ is equal to $\theta_{\mathrm{ijk}}^{l}+s_{i}^{l}$. Equation 5 aims to achieve unit standard deviation in each channel of $m^{l+1}$ and remove the effect of s, called demodulation.

Each prompt block contains 2 convolution layers, combining modulation and demodulation. Figure 2 shows that the input-specific visual prompt can be extracted from the input image after 5 prompt blocks. Then, the fused images are employed for adaptation. We adopt the additive operation to fuse the visual prompt and original image, following the same protocols [26,31]. In Eq. 2, we jointly optimize the fused image $x^{t}+\mathrm{AM}\left(x^{t}\right)$. Consequently, during the adaptation process, it is essential to use fused images to fully leverage the model’s performance.

### Adaptation

In the second stage (adaptation), we aim to optimize a target-domain model $f^{t}$. We use *g* to represent the process of the non-GAN-based methods [32–34]. The *g* process is to denoise the prediction of the source model, to obtain a pseudo-label with less wrong information. The outputs of the *g* process are pseudo-labels and confidence masks.

We denote $x^{t}$ as a sample of the target domain, AM as our well-trained AM, and $f^{s*}$ as the well-trained source model. Therefore, the softmax prediction is $f^{s*}\left(x^{t}+\mathrm{AM}\left(x^{t}\right)\right)\in {\left[0,1\right]}^{H\times W\times C}$. The pseudo-label $\hat{y}$ and confidence mask r are defined as follows:

$$\left[{\hat{y}}^{t},\;r\right]=g\left(f^{s*}\left(x+\mathrm{AM}\left(x\right)\right)\right),$$ (6)

where $r\in {\left\{0,1\right\}}^{H\times W\times 1}$ and ${\hat{y}}^{t}\in {\left[0,1\right]}^{H\times W\times C}$. Confidence mask represents if pseudo-label is confident in pixel-wise level. ${\hat{y}}^{t}$ is the denoised pseudo-label, which guides the segmentation model. If the pixel value in confidence mask is 0, those pixels in pseudo-label have a low confidence, which will not affect the segmentation model. The pseudo-label is based on the source model $f^{s*}$, and the target model $f^{t}$ is a parameter copy of $f^{s*}$ initially.

There are various methods for obtaining the pseudo-label $\hat{y}$ and confidence mask r, such as LD [32], DPL [34], and OS [33]. The most common approach for acquiring pseudo-labels and confidence masks is the double-threshold method [27,32]. To mitigate errors due to domain shift and class imbalance, we employ a category-specific thresholding method. Given softmax prediction p, the threshold $\delta^{c}$ for each category c is determined by the top k softmax value within that category:

$$\delta^{c}=\tau_{k}\left(p^{c}\right).$$ (7)

Additionally, we incorporate a global threshold λ to refine the selection of reliable pseudo-labels, ensuring robustness across all classes:

$$\hat{y}=p1\left[p>\delta^{c}\right]1\left[p>\lambda \right],$$ (8)

where 1 is the indicator function. A binary confidence mask r is applied to include only pixels with valid pseudo-labels, thereby enhancing pseudo-label efficiency and accuracy:

$$r=1\left[\sum_{c}{\hat{y}}^{c}\neq 0\right]$$ (9)

Therefore, in the second stage, the optimization process of target model $f^{t}$ can be described as follows:

$$f^{t*}=\underset{f^{t}}{\text{argmin}}E_{x^{t}∼X^{t}}\left[r\;\circ \;L_{\mathrm{CE}}\left(f^{t}\left(x^{t}+\mathrm{AM}\left(x^{t}\right)\right),\;{\hat{y}}^{t}\right)\right],$$ (10)

where ∘ means the operation of Hadamard product.

## Results

### Dataset

The proposed method is evaluated on 4 unsupervised domain adaptation tasks, including abdominal dataset and cardiac dataset, which are used by other domain adaptation studies [7,9,36,37]. The abdominal dataset is made up of 2 groups of data: 20 MRI scans from the CHAOS challenge [38], and 30 CT scans from Multi-Atlas Labeling Beyond the Cranial Vault-Workshop and Challenge [39]. Each MRI scan is a 3D volume of 256×256×L voxels, where *L* is the length of the long axis. Each CT scan is a 512×512×L 3D volume. According to the setting of [7], we divide the training set and validation set according to the ratio of 4:1. The ground truth masks are annotated as liver, right kidney, left kidney, and spleen. We proceeded with 2 domain adaptation tasks on the abdominal dataset, from CT to MRI and MRI to CT. The cardiac dataset comes from Multi-Modality Whole Heart Segmentation Challenge 2017, which contains MR and CT images [40]. The dataset consists of unpaired 20 MRI and 20 CT volumes collected at different clinical sites. Each modality was randomly split with 80% cases for training and 20% cases for testing [7]. The ground truth masks of cardiac structures include the left atrium blood cavity (LAC), the left ventricle blood cavity (LVC), the myocardium of the left ventricle (MYO), and the ascending aorta (AA). All the data were normalized as zero mean and unit variance.

### Implementation

The configuration of the source model follows [32,34] and uses DeepLab-v3 [1] segmentation model with the backbone of ResNet-50. In source model training, we employ image augmentation strategies: Blur, ShiftScaleRotate, RandomBrightnessContrast, and RandomGridShuffle. The inputs are resized to 256×256 by bilinear interpolation. The batch size is set to 4, and we adopt the Adam optimizer with 3 × 10^−5^ learning rate and 3 × 10^−5^ weight decay. All experiments were carried out with 2 NVIDIA 3090 graphic process units.

We employed 2 commonly used metrics to evaluate the segmentation performance quantitatively [7,41]. The first measurement is the Dice coefficient, calculating the volume overlap between the prediction mask and the ground truth. The second is the average surface distance (ASD) to assess the model performance at boundaries. A higher DICE and a lower ASD indicate better segmentation performance.

### Comparison experiments

As shown in Tables 1 and 2, “Source only” means that no adaptation methods are used, and the source-domain model is directly used to infer the target-domain samples. The VP-Alignment refers to the alignment process in the VP framework, where only the first stage is used without updating the source model parameters. VP-LD, VP-DPL, and VP-OS represent that different denoising methods are employed in the second stage of the VP-SFDA framework. Figure 3 shows the visualization of prediction results across different methods.

**Table 1.** Performance comparison for abdominal dataset. *P <0.05; **P <0.01; ***P <0.001

AbdominalMRI→CT	DICE ↑					ASD ↓				
	Liver	R.kid	L.kid	Spleen	Mean	Liver	R.kid	L.kid	Spleen	Mean
Source only	0.842	0.607	0.622	0.520	0.647	6.628	5.141	5.067	6.914	5.938
GAN-SFDA [42]	0.703	0.559	0.589	0.511	0.591	5.128	5.287	4.331	6.785	5.383
LD [32]	0.758	0.680	0.751	0.580	0.692	6.422	4.696	1.992	5.627	4.684
DPL [34]	0.844	0.603	0.569	0.598	0.653	4.171	2.521	2.200	3.486	3.095
OS [33]	0.835	0.607	0.570	0.620	0.658	4.677	3.228	2.242	3.809	3.489
FVP [27]	0.878	0.647	0.732	0.683	0.735	3.631	2.583	3.102	2.336	2.913
VP-Alignment	0.854*	0.667**	0.684**	0.799***	0.751***	3.936***	2.612***	3.072***	2.339***	2.990***
VP-LD	0.874***	0.690*	0.692	0.798***	0.764**	3.909***	2.587***	3.033	2.367***	2.974***
VP-DPL	0.863*	0.697***	0.691**	0.822***	0.768***	3.739*	2.551	3.153	2.249**	2.923*
VP-OS	0.889**	0.670**	0.709***	0.826***	0.773***	3.990**	2.637**	2.996**	2.220***	2.961**
AbdominalCT→MRI	DICE ↑					ASD ↓				
	Liver	R.kid	L.kid	Spleen	Mean	Liver	R.kid	L.kid	Spleen	Mean
Source only	0.582	0.789	0.686	0.009	0.517	4.661	2.678	1.545	14.518	5.850
GAN-SFDA [42]	0.521	0.812	0.712	0.448	0.623	5.188	2.589	1.414	8.779	4.493
LD [32]	0.627	0.867	0.784	0.482	0.690	4.198	2.163	1.718	6.785	3.716
DPL [34]	0.556	0.860	0.785	0.476	0.669	5.429	3.048	2.623	7.576	4.669
OS [33]	0.556	0.854	0.797	0.532	0.685	4.516	2.225	1.676	6.626	3.761
FVP [27]	0.648	0.876	0.803	0.605	0.733	4.483	2.101	1.542	6.153	3.570
VP-Alignment	0.650***	0.865**	0.785***	0.498***	0.699***	4.333**	2.233**	1.419*	6.498***	3.621***
VP-LD	0.666**	0.871*	0.822***	0.569***	0.732**	3.964**	2.271	1.455**	6.234*	3.481**
VP-DPL	0.641***	0.858*	0.801**	0.554***	0.714**	3.987**	2.094**	1.264***	6.229**	3.394**
VP-OS	0.673***	0.859*	0.776	0.561**	0.717**	4.224**	2.004**	1.187**	6.342*	3.439**

R.kid, right kidney; L.kid, left kidney

**Table 2.** Performance comparison for cardiac dataset. *P <0.05; **P <0.01; ***P <0.001.

Cardiac MRI→CT	DICE ↑					ASD ↓				
	AA	LAC	LVC	MYO	Mean	AA	LAC	LVC	MYO	Mean
Source only	0.861	0.576	0.774	0.646	0.714	12.729	11.746	6.356	4.866	8.924
GAN-SFDA [42]	0.855	0.628	0.757	0.656	0.724	13.228	9.986	7.183	4.537	8.734
LD [32]	0.878	0.734	0.814	0.725	0.788	12.754	9.450	3.758	3.490	7.363
DPL [34]	0.910	0.694	0.782	0.652	0.760	8.555	9.000	6.109	4.853	7.129
OS [33]	0.891	0.677	0.766	0.659	0.748	9.021	9.412	9.918	6.652	8.750
FVP [27]	0.856	0.719	0.795	0.640	0.753	9.012	9.003	4.374	3.520	6.477
VP-Alignment	0.885*	0.749***	0.842***	0.775***	0.813***	9.076***	8.433***	3.150***	3.560***	6.055***
VP-LD	0.920***	0.769**	0.860**	0.796**	0.836**	8.982***	8.399**	2.790**	3.272*	5.861**
VP-DPL	0.881	0.785***	0.869***	0.786***	0.830**	9.029	7.808***	2.976***	2.687***	5.625***
VP-OS	0.915**	0.774***	0.874**	0.767***	0.833***	8.431*	7.809**	2.741***	2.994***	5.494***
Cardiac CT→MRI	DICE ↑					ASD ↓				
	AA	LAC	LVC	MYO	Mean	AA	LAC	LVC	MYO	Mean
Source only	0.390	0.281	0.514	0.464	0.412	13.679	32.500	25.591	18.232	22.501
GAN-SFDA [42]	0.422	0.289	0.568	0.466	0.436	12.421	31.298	24.420	15.752	20.973
LD [32]	0.573	0.247	0.492	0.473	0.446	10.503	28.620	26.740	18.287	21.038
DPL [34]	0.574	0.249	0.514	0.508	0.461	12.478	28.551	23.827	15.522	20.094
OS [33]	0.576	0.247	0.532	0.513	0.467	11.671	28.150	24.097	15.097	19.754
FVP [27]	0.385	0.448	0.578	0.491	0.476	19.012	24.661	18.923	14.559	19.289
VP-Alignment	0.441**	0.351***	0.671***	0.618***	0.520***	11.209**	23.761***	17.934***	9.859***	15.691***
VP-LD	0.500	0.382***	0.658***	0.688***	0.557***	8.997*	23.490**	15.671***	8.429***	14.147***
VP-DPL	0.522**	0.475***	0.871***	0.706***	0.643***	9.837***	22.061***	14.382***	5.513***	12.948***
VP-OS	0.518**	0.411***	0.663***	0.694***	0.571***	11.115*	23.695**	16.381***	7.804***	14.749**

In Table 1’s “Abdominal MRI→CT” task, it is evident that VP-OS has exhibited exemplary performance. Compared to the base OS algorithm, there is a notable improvement, with the average DICE increasing from 0.658 to 0.773 and ASD ascending from 3.489 to 2.961. These enhancements are statistically significant at the P<0.01 level. Furthermore, VP-LD and VP-DPL, compared with LD and DPL, also recorded significant uplifts in the average DICE, with increases of 0.072 and 0.115, respectively. Switching to Table 2’s “Cardiac MRI→CT” task, VP-LD outshone its counterparts with an optimum average DICE of 0.836, while VP-OS achieved the best average ASD at 5.494. In the “Cardiac CT→MRI” task, VP-DPL emerged superior in both DICE and ASD metrics and was statistically significant at the P<0.001 level.

A consistent observation across various SFUDA tasks is the notable enhancement brought about by the VP-SFDA framework. In the “Cardiac MRI→CT” task, VP-LD (0.836), VP-DPL (0.760), and VP-OS (0.748) all outperformed LD (0.788), DPL (0.760), and OS (0.748) in average DICE. The VP-SFDA framework’s superiority was also mirrored in the ASD metric and other 3 SFUDA tasks. We also observed some fluctuation between different denoising methods. Specifically, in the “Abdominal MRI→CT” task, VP-OS outperformed VP-LD and VP-DPL in average DICE, while VP-DPL was superior in ASD. It became challenging to distinguish a clear rank of performance among VP-DPL, VP-LD, and VP-OS in other tasks. We attribute this phenomenon to the multiple hyperparameters associated with the DPL, LD, and OS denoising methods. Since we adhered to the hyperparameters protocol established in their respective studies [32–34], variances in performance might emerge in different SFUDA tasks. For instance, a set of hyperparameters in a particular denoising method might yield different outcomes between the CT→MRI and MRI→CT tasks. This could potentially instigate fluctuations in performance among the 3 denoising methods across various tasks. Additionally, we noticed that among the methods not utilizing the VP-SFDA framework, FVP manifested exceptional performance on the Abdominal dataset, attaining an average DICE of 0.735 (MRI→CT) and 0.733 (CT→MRI). We speculate this advantage might be attributed to FVP’s strategy of domain adaptation in the frequency domain. However, its performance was not as stellar on the cardiac dataset. We hypothesize that latent distinctions in the frequency domain characteristics between abdominal and cardiac imaging data might have substantially impacted FVP’s efficacy.

Besides, GAN-SFDA [42] is a GAN-based SFUDA framework. However, the performance shown could be better in all adaptation tasks. The reason is mainly to restore source-domain data by using the source model. The unstable training process of GAN and the lack of source data make the image generated from Gaussian noise low quality. They cannot sufficiently reflect the source-domain distribution. In addition, GAN-SFDA [42] requires a training time that is more than 5 times longer than LD [32], DPL [34], OS [33], and our proposed VP-SFDA.

Furthermore, we conducted significant difference testing across 4 unsupervised domain adaptation (UDA) tasks. We opted for the Mann–Whitney *U* test, rather than the Student’s *t* test, for the following reasons: The Student’s *t* test necessitates the fulfillment of 3 key assumptions: the independence assumption, the normality assumption, and the homogeneity of variance assumption. In our case, the medical data samples are inherently independent, thus satisfying the independence assumption. Moreover, due to the relatively small sample sizes in our dataset, the central limit theorem cannot ensures the fulfillment of the normality assumption. Besides, we found that the homogeneity of variance assumption is not met, primarily due to the close relationship between accuracy values and the models. For instance, pseudo-labels generated from prompted images tend to result in lower noise, as shown in Table 3, thereby leading to smaller variances in accuracy values. Hence, we chose to employ the Mann–Whitney *U* test, also known as the Wilcoxon rank-sum test. This nonparametric test is adept at comparing the medians of accuracy groups of 2 models, even when the assumption of variance homogeneity is not met and the number of samples is not large.

**Table 3.** Comparison of noise levels in pseudo-labels between prompted images and original images. The values in the table represent the mean square error between pseudo-label and ground truth.

Type	abd. M2C	abd. C2M	car. M2C	car. C2M	Mean
Source only	0.190	0.213	0.186	0.247	0.209
VP-Alignment	0.123	0.149	0.128	0.177	0.144

Our aim was to rigorously validate the significant differences between VP-SFDA and the compared methods. Therefore, we conducted significant difference tests between methods employing the VP-SFDA framework and those that did not. For example, we compared VP-LD with LD, VP-DPL with DL, and VP-Alignment with Source only in Tables 1 and 2. In our experimental results, we observed that the VP-SFDA framework exhibited remarkably competitive performance when compared to existing state-of-the-art SFUDA methods across 4 diverse tasks. Specifically, we aimed to statistically validate the significance of VP-SFDA’s performance improvements relative to the compared methods. In the abdominal MRI→CT task, we observed a notable enhancement in DICE scores for LD, DPL, and OS methods when integrated into the VP-SFDA framework. The DICE scores increased from 69.2%, 65.3%, and 65.8% to 76.4%, 76.8%, and 77.2%, respectively. Importantly, these improvements were found to be statistically significant at the P<0.01 level.

**Fig. 4.**
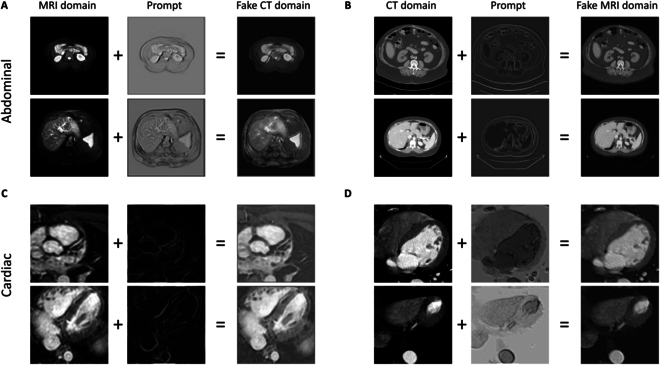
Visualization of alignment prompt. (A and B) Samples of abdominal dataset. (C and D) Samples of cardiac dataset. (A and C) CT→MRI adaptation task. (B and D) MRI→CT adaptation task.

In Fig. 4, we visualize the alignment prompt and the fusion of the prompt and original image.

### Noise experiments

In the noise analysis experiments, we directly compared the noise levels in pseudo-labels generated from original and prompted images, without applying any denoising techniques (LD, DPL, and OS). Specifically, “Source only” in Table 3 represents the setup where pseudo-labels are generated directly from original images, while “VP-Alignment” represents the setup using prompted images. We calculated the mean square error (MSE) between the pseudo-labels and the manually annotated ground-truth as a measure of noise:

$$\mathrm{mse}=\frac{1}{C}\sum_{c=0}^{C}{\left({\hat{y}}_{c}-y_{c}\right)}^{2},$$ (11)

where ${\hat{y}}_{c}$ represents the pseudo-label for category c and $y_{c}$ represents the corresponding ground truth. C is the category number, which is 4 in our study.

The results from experiments reveal a clear trend across all 4 tasks. The average noise level in pseudo-labels generated from prompted images is lower, with an MSE of 0.144, compared to pseudo-labels generated from original images, which exhibit a higher noise level with an MSE of 0.209.

Furthermore, we also validated this observation through segmentation experiments. For instance, as shown in Table 1, in the context of the abdominal MRI→CT task, training a segmentation model with pseudo-labels derived from prompted target-domain samples yields a DICE performance score of 0.751. In contrast, segmentation models trained without the use of prompts achieve a lower DICE performance score of 0.647 in the same task. Similar results have been observed in other tasks.

These quantitative results provide strong support for our assertion that the pseudo-labels generated from prompted images exhibit reduced noise levels compared to those generated from original images.

### Ablation experiments

The ablation study is bifurcated into 2 integral components: a comparative analysis of visual prompt patterns and an examination of the AM’s structure. The outcomes of the former are illustrated in Table 4, while the results of the latter are delineated in Table 5. The input-agnostic prompt is generated by iteratively optimizing parameters. Specifically, the prompt is conceptualized as having a resolution *W* width and *H* height, where each pixel or unit in this grid represents a trainable parameter. For a prompt of this resolution, there are *WH* trainable parameters in total. In our implementation, these parameters are optimized through a backpropagation process and the same loss function as the input-specific prompt (AM).

**Table 4.** Ablation experiments of input-agnostic and input-specific on 4 adaptation tasks

Abdominal MRI→CT	DICE					ASD				
	Liver	R.kid	L.kid	Spleen	Mean	Liver	R.kid	L.kid	Spleen	Mean
Input-agnostic	0.840	0.608	0.624	0.545	0.654	4.875	3.096	3.152	4.826	3.988
Input-specific (ours)	0.854	0.667	0.684	0.799	0.751	3.936	2.612	3.072	2.339	2.990
AbdominalCT→MRI	DICE					ASD				
	Liver	R.kid	L.kid	Spleen	Mean	Liver	R.kid	L.kid	Spleen	Mean
Input-agnostic	0.628	0.823	0.713	0.021	0.546	4.206	1.961	1.564	13.741	5.368
Input-specific (ours)	0.640	0.855	0.775	0.488	0.689	4.533	2.433	1.619	6.698	3.821
Cardiac MRI→CT	DICE					ASD				
	AA	LAC	LVC	MYO	Mean	AA	LAC	LVC	MYO	Mean
Input-agnostic	0.862	0.584	0.775	0.638	0.715	12.605	11.186	6.195	4.771	8.690
Input-specific (ours)	0.885	0.749	0.842	0.775	0.813	9.076	8.433	3.150	3.560	6.055
Cardiac CT→MRI	DICE					ASD				
	AA	LAC	LVC	MYO	Mean	AA	LAC	LVC	MYO	Mean
Input-agnostic	0.446	0.287	0.524	0.473	0.432	13.980	29.885	24.564	18.379	21.702
Input-specific (ours)	0.441	0.351	0.671	0.618	0.520	11.209	23.761	17.934	9.859	15.691

**Table 5.** Ablation experiments conducted on AMs with varying structures. The terms “abd.” and “car.” represent the abdominal dataset and cardiac dataset, respectively. “M2C” and “C2M” correspond to the “MRI→CT” and “CT→MRI” tasks, respectively. The values in the table represent the average Dice scores across multiple categories for each task.

Structure	abd. M2C	abd. C2M	car. M2C	car. C2M	Mean
Plain	0.723	0.671	0.785	0.502	0.670
PB (*n* = 5)	0.751	0.699	0.813	0.520	0.696
PB (*n* = 2)	0.680	0.650	0.742	0.469	0.635
PB (*n* = 3)	0.728	0.678	0.786	0.504	0.674
PB (*n* = 4)	0.743	0.692	0.804	0.514	0.688
PB (*n* = 5)	0.751	0.699	0.813	0.520	0.696
PB (*n* = 6)	0.737	0.691	0.820	0.510	0.690
PB (*n* = 7)	0.732	0.694	0.806	0.505	0.684
PB (*n* = 10)	0.723	0.681	0.793	0.495	0.673

Table 4 underscores the performance disparity between our proposed input-specific pattern and the conventional input-agnostic pattern across 4 SFUDA tasks. In the context of the abdominal MRI→CT task, the input-specific pattern exemplifies its superior efficacy by attaining an average DICE of 0.751 across Liver, Spleen, and 2 Kidney categories, markedly outpacing the 0.654 achieved by the input-agnostic approach. A similar trend is observable in the CardiacMRI→CT task, where the input-specific pattern achieves an average DICE of 0.813, transcending the 0.715 for the input-agnostic counterpart. The efficacy of the input-specific paradigm is consistently manifested across all 4 SFUDA tasks. This preeminence underscores the augmented adaptation capacity of the input-specific visual prompt, affirming its enhanced suitability for SFUDA tasks in comparison to the input-agnostic visual prompt. The nuanced design of the input-specific pattern, tailored to individual data intricacies, empowers it with a refined adaptability, ensuring a more accurate and efficient domain adaptation.

As shown in Table 5, we compared 2 types of networks: “plain”, representing a basic CNN convolutional network, and “PB”, which incorporates prompt blocks designed as illustrated in Fig. 2. The n parameter denotes the number of prompt blocks used. We conducted this comparison across 4 distinct tasks.

Our experiments revealed that the “plain” CNN network did not perform as effectively as the PB network, which utilized the same number of layers but integrated the prompt blocks. Regarding the selection of the number of prompt blocks (*n*), we conducted a thorough analysis to determine its impact on performance. Based on our findings, we observed that there was a performance improvement when the number of prompt blocks was increased, specifically when the count was less than 5. However, as the number of blocks continued to increase beyond 5, we observed diminishing returns. In fact, when the block count reached 10, there was a decrease in performance, as indicated by the Dice score of 0.673. Ultimately, our experiments demonstrated that utilizing 5 prompt blocks yielded the best results, with an average Dice score of 0.696.

### Parameter quantity

Prior works on source-free domain adaptation for medical image segmentation, such as LD [32], DPL [34], and OS [33], leverage the widely used DeepLabV3 architecture [1] with ResNet50 as the backbone, which has approximately 3.5 million trainable parameters. These methods update the parameters of the source segmentation model during the adaptation process so that their parameters in Table 6 are 3.5 million. GAN-SFDA [42] requires training a GAN, leading to additional parameters. However, upon reviewing Tables 1 and 2, it becomes evident that the effectiveness of GAN-SFDA is relatively suboptimal. This limitation might stem from the integration of an unstable adversarial training mechanism. In the context of SFUDA tasks, the unavailability of source-domain data exacerbates the difficulty of adversarial training, thereby undermining the model’s effectiveness and performance.

**Table 6.** Number of parameters for different methods

Method	Parameters	Method	Parameters
SFDA [42]	97.6M	VP-Alignment	3.5M
LD [32]	39.6M	VP-LD	39.6M
DPL [34]	39.6M	VP-DPL	39.6M
OS [33]	39.6M	VP-OS	39.6M

Our proposed approach, VP-SFDA, contains 2 stages. For the first stage (prompting), we need to update the AM while freezing the parameters of the source model. Therefore, the number of parameters for the AM is 3.5 million. The second stage involves fixing the AM’s parameters and updating the parameters of the target model, which has the same structures as the source model. Consequently, the trainable parameters of VP-LD, VP-DPL, and VP-OS are the same as those of LD, DPL, and OS, respectively. Since VP-LD, VP-DPL, and VP-OS incorporate the AM, their total parameter count amounts to 43.1 million, which is composed of 39.6 million trainable parameters and 3.5 million frozen parameters.

## Discussion

### The combination of visual prompt and denoised pseudo-labels is better

Our 2-stage framework, which leverages denoised pseudo-labels, offers substantial advantages over methods like ProSFDA [31] that rely solely on visual prompts. First, our approach inherently includes the visual prompting process. Even in scenarios where denoised pseudo-labels might be less effective, our model is capable of achieving at least equivalent performance to methods that utilize only visual prompts. Second, methods like ProSFDA [31], which do not modify the source model and only use visual prompt, tend to suffer from poor robustness. The inference model only trained on source-domain data, making them vulnerable to performance degradation when encountering input data variations. In contrast, our method not only utilizes visual prompts to bridge domain discrepancies but also enhances the source model’s robustness through the fine-tuning process with denoised pseudo-labels. This fine-tuning involves data from both the source and target domains, thereby enriching the training dataset and subsequently enhancing the model’s robustness.

### The comparison of the input-agnostic prompt and the input-specific prompt

**Fig. 5.**
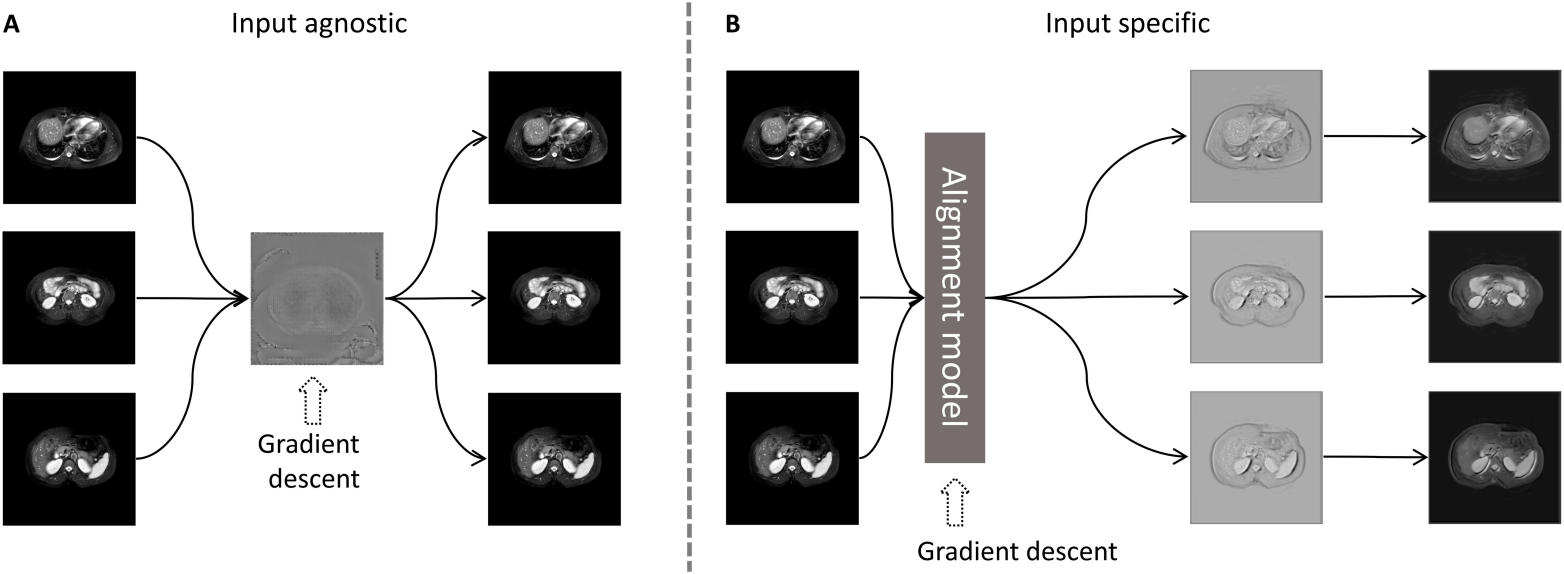
Comparison of input-agnostic and input-specific. (A) The input-agnostic prompt is a prompt for an entire domain. (B) The input-specific prompt is one prompt for one image.

There are 2 reasons. First, as shown in Fig. 5A, input-agnostic visual prompts apply a uniform pattern or style to all incoming data, treating all inputs the same. It might not capture the nuanced differences between individual data samples. As it is a “one-size-fits-all” prompt, it may not be optimized for specific characteristics of individual data samples. This can limit the performance of the domain adaptation process. Second, input-agnostic visual prompt has only been validated for effectiveness in 2-dimensional segmentation tasks of the OC/OD [31]. However, the adaptation challenges are greater in 3-dimensional segmentation tasks involving CT and MR data under unsupervised domain adaptation (UDA) settings. In this more complex scenarios, input-agnostic approach was ineffective, as shown in Table 4. In contrast, input-specific visual prompts in Fig. 5B show enhanced adaptability and performance in these challenging settings.

### The expectations of VP-SFDA framework

There are 3 expectations from our framework. First, by effectively bridging the gap between different imaging modalities, our model is expected to improve the accuracy of diagnostic algorithms when applied to varied data, ensuring high reliability of medical imaging analysis across different machines and settings. Second, reducing the need for manual annotation and retraining of models for different modalities lowers the costs associated with deploying artificial intelligence (AI) in medical imaging. Third, since the model adapts without requiring direct access to the original training data, it supports compliance with stringent data privacy laws and regulations, which is crucial for adoption in various global regions. Moreover, we have the following technical contributions:

1. Proposed a new two-stage SFUDA framework (VP-SFDA). In the first stage, our designed AM creates visual prompts to effectively shorten the domain shift. In the second stage, we apply denoising methods to further improve performance.

2. Developed a novel input-specific pattern. This pattern adapts images from the target domain to align with the source-domain distribution, catering to the individual characteristics and features of each data sample.

## Conclusion

This paper proposes a novel 2-stage framework for SFUDA called VP-SFDA. Our method utilizes input-specific visual prompts and batch normalization constraint to enable the AM to learn domain-specific knowledge and align the target-domain data with the source-domain distribution in the first stage. Input-specific pattern caters to the individual characteristics and features of each data sample, which achieves better adaptation capability. In the second stage, we adopt denoising methods to improve the performance further. We demonstrated the effectiveness of VP-SFDA through comparative experiments on 4 medical SFUDA tasks. The prompting performance of VP-SFDA has surpassed other methods, and the second stage can further improve its performance. In addition, ablation experiments demonstrated the advantages of domain-specific pattern. We hope that the proposal of the VP-SFDA can promote the consideration and development of privacy in the medical imaging domain adaptation issue.

We outline 2 key avenues for further enhancing the VP-SFDA framework: First, expanding the framework to universal body segmentation model could broaden its applicability across various medical imaging tasks, such as brain region segmentation. We also plan to investigate the scalability of VP-SFDA by applying it to larger datasets and across more diverse medical imaging modalities.

## Ethical Approval

This study uses publicly available datasets and does not involve any human or animal subjects; therefore, ethical approval is not required.

## Data Availability

The data supporting this study are from multiple sources. First, the CHAOS Challenge dataset is accessible at https://chaos.grand-challenge.org/. Second, data from the “Multi-Atlas Labeling Beyond the Cranial Vault - Workshop” are available at https://www.synapse.org/#!Synapse:syn3193805/wiki/89480. Lastly, the “Multi-Modality Whole Heart Segmentation” dataset, offering detailed heart segmentation modalities, can be found at https://zmiclab.github.io/zxh/0/mmwhs/.
