# Supplementary material for: VP-SFDA: Visual Prompt Source-Free Domain Adaptation for Cross-Modal Medical Image
Source: Health Data Sci. 2025 Jan 7;5:0143. doi: 10.34133/hds.0143 (PMC12063703; doi:10.34133/hds.0143)

Health
Data Science
A SCIENCE PARTNER JOURNAL

Permission for Photographic/ Illustration Use

I hereby grant to Peking University Health Science Center ("PKUHSC") a perpetual and irrevocable non-exclusive right to use and authorize others to use the photo/illustration/image/figure below (the "Material(s)") in connection with an article on

VP-SFDA: Visual Prompt Source-Free Domain Adaptation for Cross-Modal Medical Image
____
(the "Article") to be published and distributed by PKUHSC and its licensees/ assigns in an issue of *Health Data Science*, in any and all media in which the Article and issue may be published or distributed, and for any later use by PKUHSC of that Article in any format or medium now known or hereafter developed, including Web-based publishing.

I warrant that I am the owner and/or authorized representative of the owner of the copyright in the Material(s) and that I have the authority to grant PKUHSC the permission and rights granted herein. I further warrant that the Material(s) and use thereof does not and will not violate the copyright or other personal or proprietary right of any person.

Authorization

Full Name: Zhaoheng Xie

Signature: Zhaoheng Xie 数字签名者：Zhaoheng Xie 日期：2024.08.18 15:27:50 +08'00'

* Attach images on page 2 before signing

Date: 08/18/2024

{00153802;v1}

Images covered by this permission:

Filename: figure1.png

Name to appear in credit line: Zhaoheng Xie

INSERT THUMBNAIL IMAGE OF MATERIAL(S)

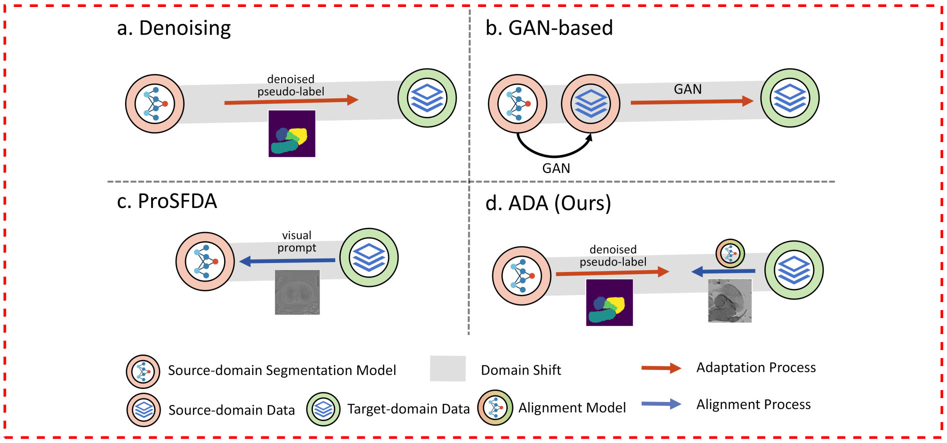

Images covered by this permission:

Filename: figure2.png

Name to appear in credit line: Zhaoheng Xie

INSERT THUMBNAIL IMAGE OF MATERIAL(S)

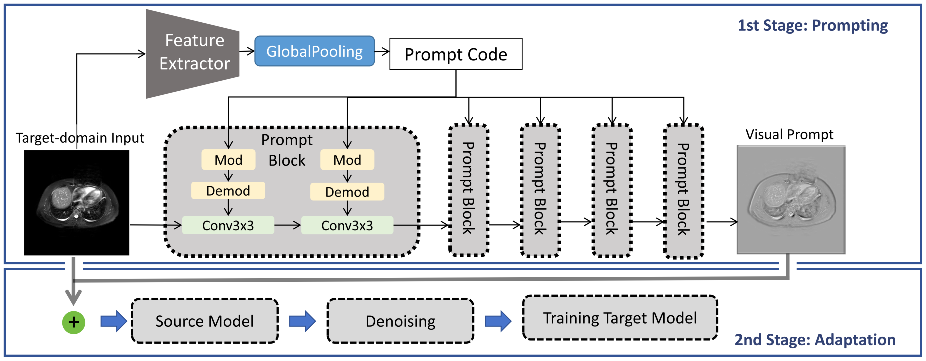

{00153802;v1}

Images covered by this permission:

Filename: figure3.png

Name to appear in credit line: Zhaoheng Xie

INSERT THUMBNAIL IMAGE OF MATERIAL(S)

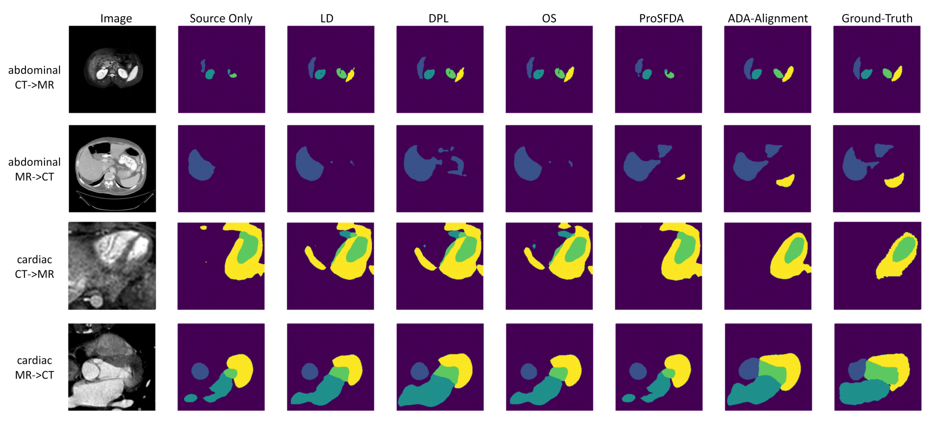

Images covered by this permission:

Filename: figure4.png

Name to appear in credit line: Zhaoheng Xie

INSERT THUMBNAIL IMAGE OF MATERIAL(S)

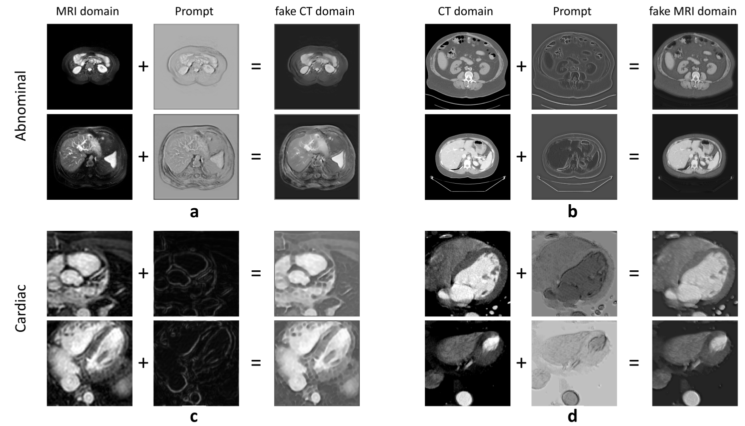

Images covered by this permission:

Filename: figure5.png

Name to appear in credit line: Zhaoheng Xie

INSERT THUMBNAIL IMAGE OF MATERIAL(S)

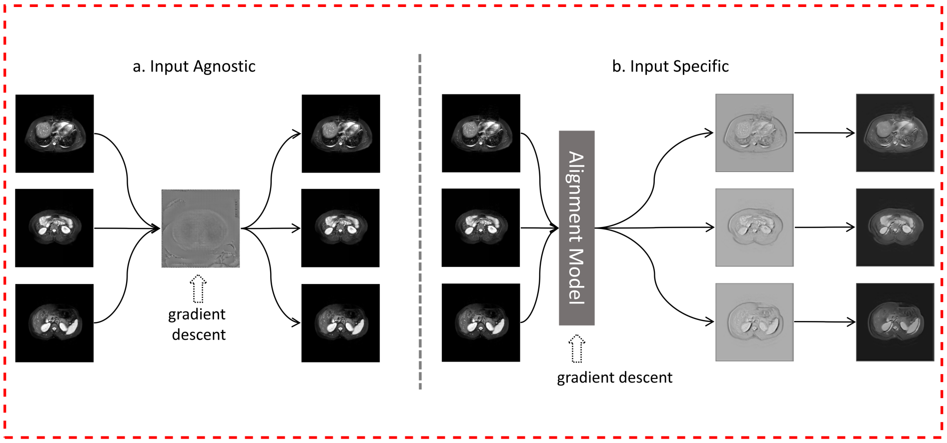

Supplement: Supplementary 1 — Figs. S1 to S5 [file hds.0143.f1.zip › HDS_ImagePermission_v2.pdf]
